# Honeycomb Boron on Al(111): From the Concept of Borophene to the Two-Dimensional Boride

**DOI:** 10.1021/acsnano.1c05603

**Published:** 2021-08-30

**Authors:** Alexei B. Preobrajenski, Andrey Lyalin, Tetsuya Taketsugu, Nikolay A. Vinogradov, Alexander S. Vinogradov

**Affiliations:** † MAX IV Laboratory, Lund University, 22100 Lund, Sweden; ‡ Institute for Chemical Reaction Design and Discovery (WPI-ICReDD), Hokkaido University, Kita 21 Nishi 10, Sapporo 001-0021, Japan; § Center for Green Research on Energy and Environmental Materials (GREEN), National Institute for Materials Science, Namiki 1-1, Tsukuba 305-0044, Japan; ⊥ Department of Chemistry, Faculty of Science, Hokkaido University, Kita 10 Nishi 8, Sapporo 060-0810, Japan; ∥ St. Petersburg State University, St. Petersburg, 198504, Russia

**Keywords:** borophene, 2D materials, aluminum boride, STM, XPS, NEXAFS, DFT

## Abstract

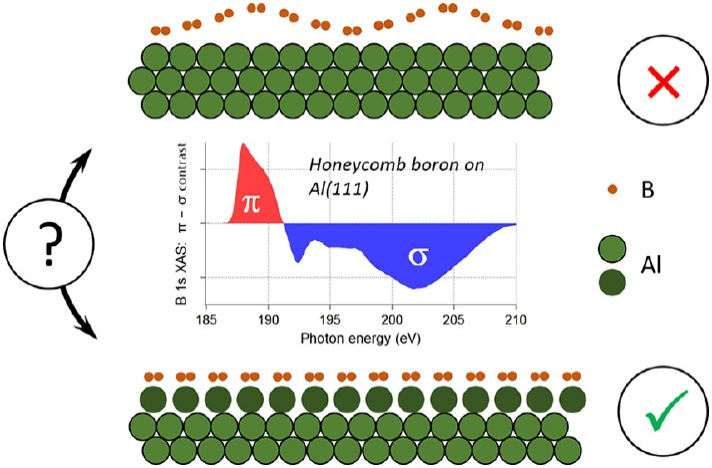

A great variety of two-dimensional (2D) boron allotropes (borophenes) were extensively studied in the past decade in the quest for graphene-like materials with potential for advanced technological applications. Among them, the 2D honeycomb boron is of specific interest as a structural analogue of graphene. Recently it has been synthesized on the Al(111) substrate; however it remains unknown to what extent does honeycomb boron behave like graphene. Here we elucidate the structural and electronic properties of this unusual 2D material with a combination of core-level X-ray spectroscopies, scanning tunneling microscopy, and DFT calculations. We demonstrate that in contrast to graphene on lattice-mismatched metal surfaces, honeycomb boron cannot wiggle like a blanket on Al(111), but rather induces reconstruction of the top metal layer, forming a stoichiometric AlB_2_ sheet on top of Al. Our conclusions from theoretical modeling are fully supported by X-ray absorption spectra showing strong similarity in the electronic structure of honeycomb boron on Al(111) and thick AlB_2_ films. On the other hand, a clear separation of the electronic states of the honeycomb boron into π- and σ-subsystems indicates an essentially 2D nature of the electronic system in both one-layer AlB_2_ and bulk AlB_2_.

## Introduction

Elemental boron is a material famous for its rich phase diagram,^1^ with the majority of stable bulk phases composed of interlinked icosahedral B_12_ clusters. This polymorphism is a direct consequence of interplay between directional and multicenter bonding, as boron cannot form crystalline solids with only two-center covalent bonds due to its electron deficiency. Stimulated by the discovery of fascinating electronic properties in graphene, an intense search for two-dimensional (2D) forms of boron started in a quest for unknown phenomena. In contrast to graphene and hexagonal BN, a large variety of 2D boron sheet allotropes (called borophenes) were predicted in a freestanding form^2−4^ and supported.^5,6^ Essentially, any borophene can be considered as a triangular B lattice with either (less energetically favorable) buckling or (more energetically favorable) periodic vacancies (hollow hexagons, HH) in different motifs and concentrations.^2^ For purely planar freestanding borophene this vacancy concentration can vary from 0 for a triangular sheet with no HH to 1/3 for a hexagonal (honeycomb or graphene-like) sheet, with values between 1/9 and 2/15 for the most stable structures.^3^

Experimentally it has been demonstrated that borophene with different motifs can be formed on metal substrates, including Ag(111),^7,8^ Cu(111),^9^ Al(111),^10^ Ir(111),^11^ and others. The nature of boron–substrate interaction determines the amount of electron charge donated to borophene. Therefore, by selecting a suitable metal substrate one can in principle regulate the charge transfer and therefore the concentration of HH. As HH are electron-deficient sites prone to accept electrons,^2−4^ the larger the net negative charge transfer to the boron sheet, the less dense it becomes. As an extreme case, purely honeycomb boron (HB), consisting of only void B honeycombs and possessing therefore the least density of the reported borophene polymorphs, can be stabilized by the Al(111) substrate due to very large transfer of nearly one electron per B atom from the substrate.^10^

In the rich family of 2D boron sheets, HB is particularly interesting for its structural similarity to graphene. Although HB is highly unstable in its free-standing form,^2,12,13^ its stability on Al(111) makes it a viable object not only for theoretical but also experimental studies. In particular, the electronic structure of sp^2^-bonded boron and sp^2^-bonded carbon can be compared directly by using different spectroscopic techniques. In this context, angle-dependent NEXAFS (near-edge X-ray absorption fine structure) spectroscopy can be especially helpful, as it may allow probing π- and σ-electronic subsystems in borophene separately. Also, it is highly interesting to compare experimentally electronic and structural properties of HB on Al(111) with those in the closely related prototypical bulk layered compound AlB_2_, where flat hexagonal Al sheets alternate with flat honeycomb B sheets. This, in combination with large-scale calculations, can help to reveal the exact atomic arrangement of HB on Al(111) and the nature of observed triangular corrugation,^10^ which remains unclear. Recently a study of the electronic structure of HB on Al(111) by angle-resolved photoemission spectroscopy (ARPES) revealed several Dirac cones in the band structure, which were interpreted as signatures of an AlB_2_ surface layer weakly bonded to the underlying Al(111) substrate.^14^ On the other hand, a large difference in the B–Al layer separation and the B adhesion energy between favorable (fcc-hcp) and unfavorable (top-fcc and top-hcp) adsorption sites^15^ may be indicative of a corrugated HB sheet, somewhat similar to graphene on lattice-mismatched substrates.^16^

In this paper, we perform a comparison of the structure, growth processes, and electronic properties of HB on Al(111) with thin films of AlB_2_ grown on the same substrate and studied by means of synchrotron-based X-ray photoemission spectroscopy (XPS), NEXAFS spectroscopy, scanning tunneling microscopy (STM), low-energy electron diffraction (LEED), and density-functional theory (DFT) calculations. (Although epitaxial films of AlB_2_ may slightly deviate structurally from the parent bulk compound, we refer to the (−B–Al−)_*n*_/Al(111) samples as AlB_2_ films throughout this article based on our interpretation of the data.) We reveal a transition from HB on Al(111) to AlB_2_ and suggest a model for the HB arrangement on Al(111). Furthermore, we demonstrate experimentally a separation of unoccupied states in HB on Al(111) into B 2p(π*)- and 2p(σ*)-electron subsystems and compare details of electronic structure in HB with those in graphene and other related compounds.

## Results and Discussion

**Figure 1:**
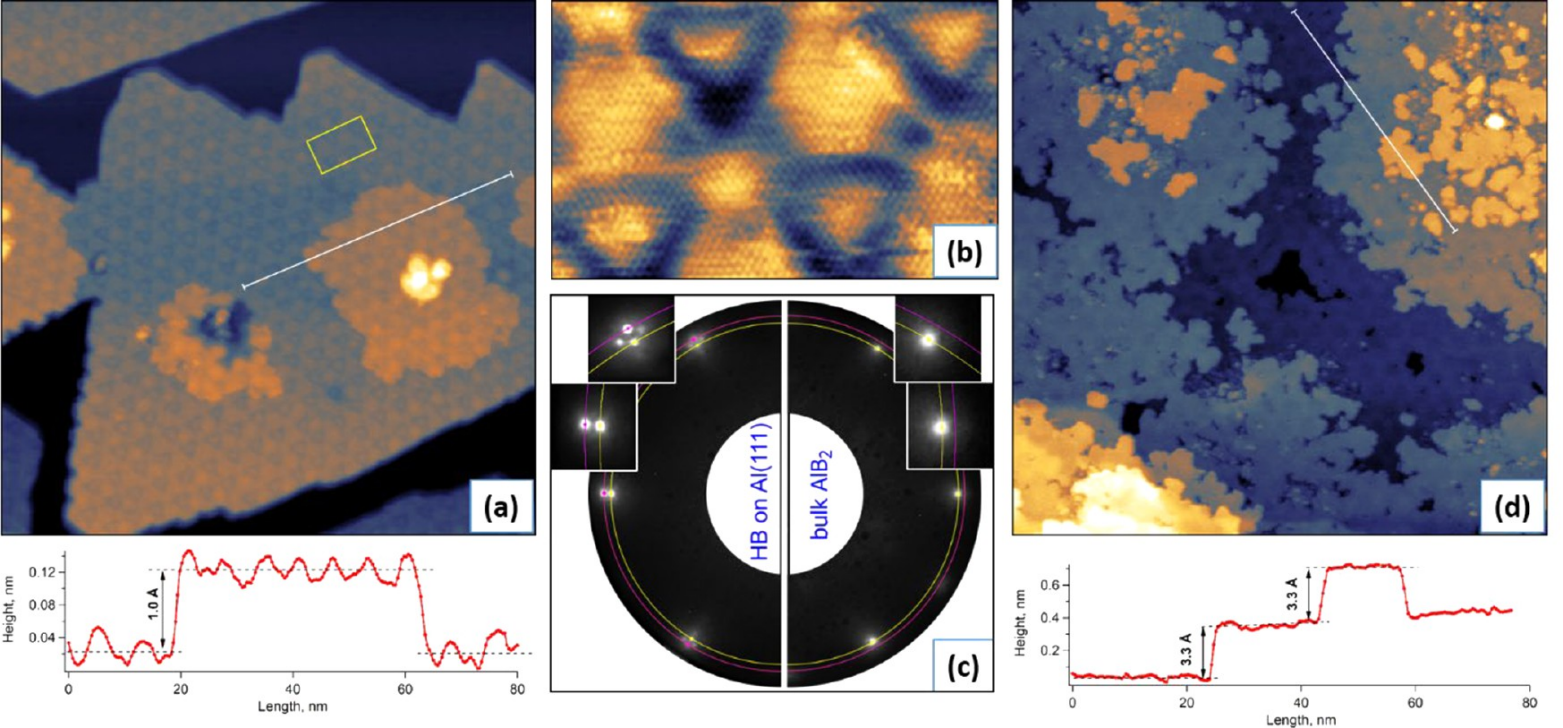
STM and LEED for HB and bulk AlB_2_ on Al(111). (a) 150 × 150 nm^2^ STM image from a sample with nominal coverage of 0.5 ML HB. Note that on top of the HB islands with a characteristic triangular superstructure some other islands with irregular shape are growing. Bottom panel: height profile along the white line in the image. (b) 15 × 9 nm^2^ STM image showing one triangular supercell outlined by a yellow rectangular in (a) in high resolution. (c) LEED patterns taken at 48 eV from a sample with sub-ML HB coverage (left, with insets zooming in on the moiré spots) and 3 ML nominal HB coverage (right, with insets zooming in on the AlB_2_ spots). Original Al(111) spots corresponding to the lattice parameter of 2.86 Å are marked with pink dots on a pink circle; moiré spots corresponding to a lattice parameter of 2.98 Å are marked with yellow dots on a yellow circle. (d) 150 × 150 nm^2^ STM image from a sample with nominal coverage of 3 ML HB and reflecting the morphology of bulk AlB_2_ film on Al(111). Bottom panel: height profile along the white line in the image. STM parameters (sample bias/tunnelling current) are −1.0 V/340 pA (a), 0.01 V/20 pA (b), and −1.5 V/280 pA (d).

In full agreement with the previous reports,^10,14^ deposition of sub-ML quantities of boron on Al(111) at a substrate temperature around 180 °C results in the formation of HB islands decorated by a typical triangular superstructure, as can be seen in the STM images of Figure 1a and b. This moiré superstructure is reflected also in the LEED pattern as extra spots surrounding the original Al(111) spots (left part of Figure 1c). From the LEED pattern analysis we conclude that the mismatch between lattice constants of Al(111) and the HB-induced structure is 4.2 ± 0.2%. Therefore, the lattice constant of this surface structure is *a*_S_ = 0.298 nm (assuming the bond length in Al(111) is *a*_Al_ = 0.286 nm), and, consequently, 24 *a*_S_ are matching 25 *a*_Al_, forming a 24:25 supercell with a period of 7.15 nm. These numbers are similar to those in the paper by W. Li *etal*.^10^ and identical to those in the paper by D. Geng *etal*.^14^

Single-monolayer (ML) HB with triangular superstructures has a tendency for arranging in triangular or hexagonal islands on Al(111) at sub-ML boron coverage. Although these islands are clearly dominating the surface composition, they are not the only ones, as can be seen in Figure 1a. Some other patches with irregular shape start to appear on top of single-ML HB islands, close to their centers. Despite many attempts, we did not manage to grow a sample with substantial HB coverage without these patches, and the area covered by them was typically around 20% of the total coverage. These extra islands have the same triangular superstructure as the single-layer HB and often have some loose boron clusters on top. A plausible interpretation of these structures is the following: when boron atoms or clusters are landing in a center of a large HB island, they start to migrate toward its edge, but get trapped on a defect and start to intercalate. As the expected stable structure in this case is the layered compound AlB_2_, the intercalated boron penetrates probably between the first and second Al sheets, thus forming an island of an AlB_2_ layer underneath HB on top. This process is expected to elevate the top Al layer by 0.96 Å, a difference in separation between Al sheets along the [111] direction in metallic Al (2.34 Å) and in AlB_2_ (3.27 Å). As can be seen in the height profile across an “extra” island (at the bottom of Figure 1a), the measured value of its elevation is indeed close to 1 Å. From the same profile in Figure 1a it can also be seen that the height of oscillation across the supercell is similar on the single-layer HB island and on the elevated “extra” island, close to 50 pm.

In the following, we refer to this stacking of layers (HB-Al-HB-Al-bulk Al) as “two-layer AlB_2_”; embedding of one more HB layer would result in “three-layer AlB_2_”, *etc*. This notation implies that areas with single-layer HB on Al(111) can be formally referred to as “one-layer AlB_2_”; in the next section we will show that this formal notation is actually perfectly relevant.

Upon boron coverage exceeding nominally 1 ML HB, not only the LEED spots from Al(111) but also moiré-related spots are fading away, while the spots from AlB_2_ are growing in intensity. At the nominal thickness above 3 ML of HB (*i*.*e*., three-layer AlB_2_) the spots of AlB_2_ become strongly dominating the LEED pattern (right part of Figure 1c). The surface morphology revealed by STM at this coverage is shown in Figure 1d. Apparently, it represents a collection of randomly shaped AlB_2_ islands of different thickness. This randomness and relatively small size of islands are reflected also in a broadening of the principal AlB_2_ spots in LEED (insets in the right part of Figure 1c). The two-layer AlB_2_ areas (like the one in the middle of Figure 1d) retain the supercell motif with triangular corrugation typical for HB on Al(111), while at larger thickness this characteristic moiré pattern is gradually fading away (see also Figure S1). The white line in Figure 1d is drawn along the areas of two-layer → three-layer → four-layer → three-layer of AlB_2_, as can be seen from the corresponding height profile, where the steps are 3.3 Å high, corresponding to the lattice constant of 3.27 Å in bulk AlB_2_.

**Figure 2:**
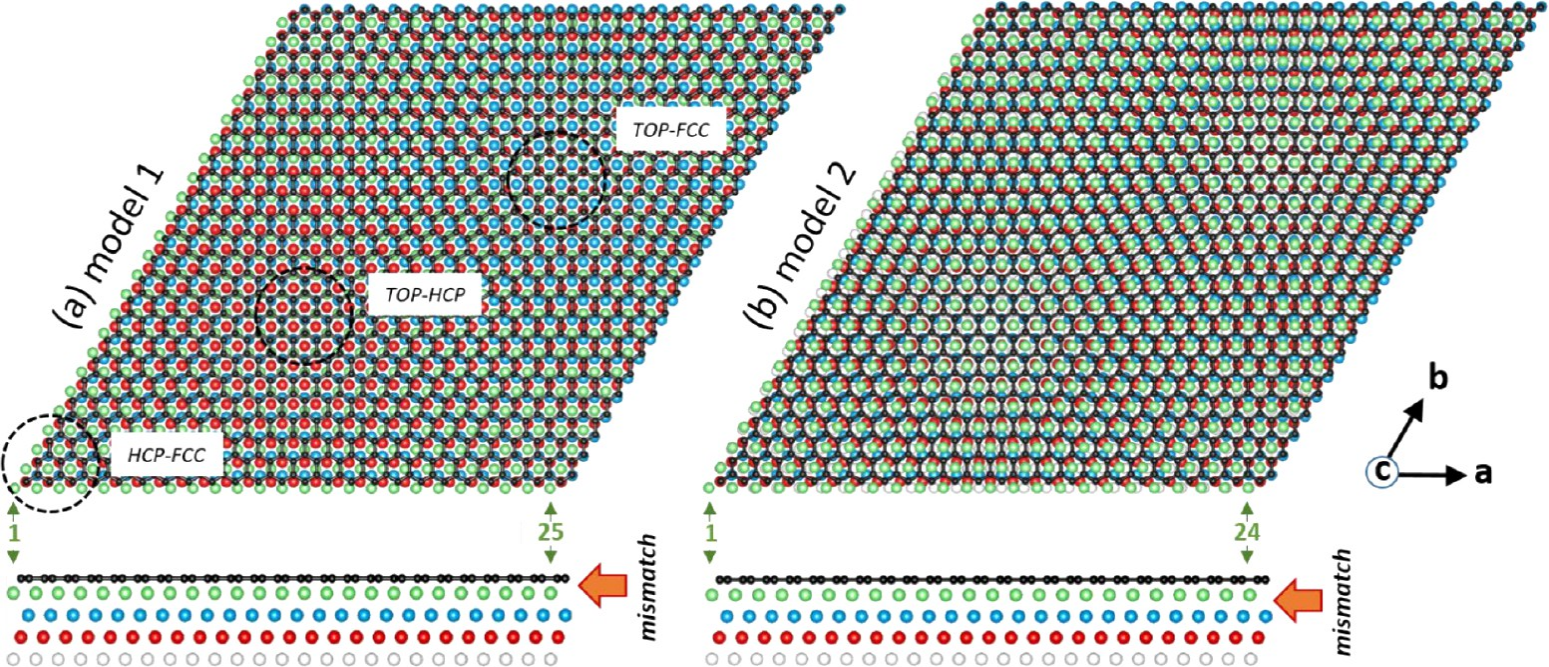
Two possible realizations of the 24:25 supercell at the interface between honeycomb borophene and Al(111). For each model, two projections are shown: along vector **c** (top) and along vector **a** (bottom). B atoms are shown in black; Al atoms in the top, second, third, and fourth layers are shown in green, blue, red, and white, respectively. In model 1 (a) the top Al layer has 25 × 25 atoms in the supercell; it is lattice-matched to other Al layers but mismatched relative to the HB lattice. Areas with predominantly hcp-fcc, top-hcp, and top-fcc boron adsorption sites are circled and labeled. In model 2 (b) the top Al layer has 24 × 24 atoms in the supercell; it is lattice-mismatched to other Al layers but matched relative to the HB lattice. Numbers (in green) are counting Al atoms in the top layer along vector **a**.

Although the overall process of AlB_2_ growth on Al(111) is fairly understandable, the exact structure of HB on Al(111) remains an open question. The observed 24:25 supercell can be realized in two main structural motifs, as illustrated in Figure 2:

model 1, where (24 × 24) HB unit cells are matching (25 × 25) unit cells of Al(111) directly, and
model 2, where HB is lattice-matched to the top Al layer forming a one-layer AlB_2_, and then (24 × 24) unit cells of this AlB_2_ layer are matched to the (25 × 25) unit cells of Al(111).

The only difference between these models is the density of the top Al layer: in model 1 it is the same as in all other Al layers (25 × 25 atoms in the supercell), while in model 2 it is slightly less (24 × 24 atoms in the supercell). If model 1 is true, the top HB layer is expected to corrugate slightly above the top Al layer to accommodate the mismatch with Al(111). This behavior is similar to corrugation of graphene on lattice-mismatched metal surfaces, which tends to ripple like an elastic blanket even on strongly interacting substrates such as Ru(0001).^27^ Model 2 has been recently proposed by D. Geng and co-workers,^14^ based on the conclusion that DFT calculations for a freestanding one-layer AlB_2_ give a better match to the experimental lattice constant than for a freestanding HB. However, a freestanding HB is a hypothetical object and can hardly exist in nature. Therefore, to accurately describe an atomic arrangement at the interface, it is essential to compare not freestanding systems but rather 1 ML of HB on Al(111) with one-layer AlB_2_ on Al(111), that is, model 1 *versus* model 2.

A geometry optimization was performed by DFT calculations on both models shown in Figure 2. In each case, four layers of Al atoms were included in the slab, with fixed geometry for the two bottom layers; positions of the two upper layers and the HB layer were allowed to relax. The vacuum spacing above HB was set to 0.94 nm. Not surprisingly, the geometry optimization calculations were computationally intense, as the total number of atoms in the slab was 3603 and 3652 for models 1 and 2, respectively. For model 1 we additionally had to calculate a slab with five layers of Al atoms (three relaxed and two fixed) to investigate how geometry optimization is converging with an increasing number of metal layers.

**Figure 3:**
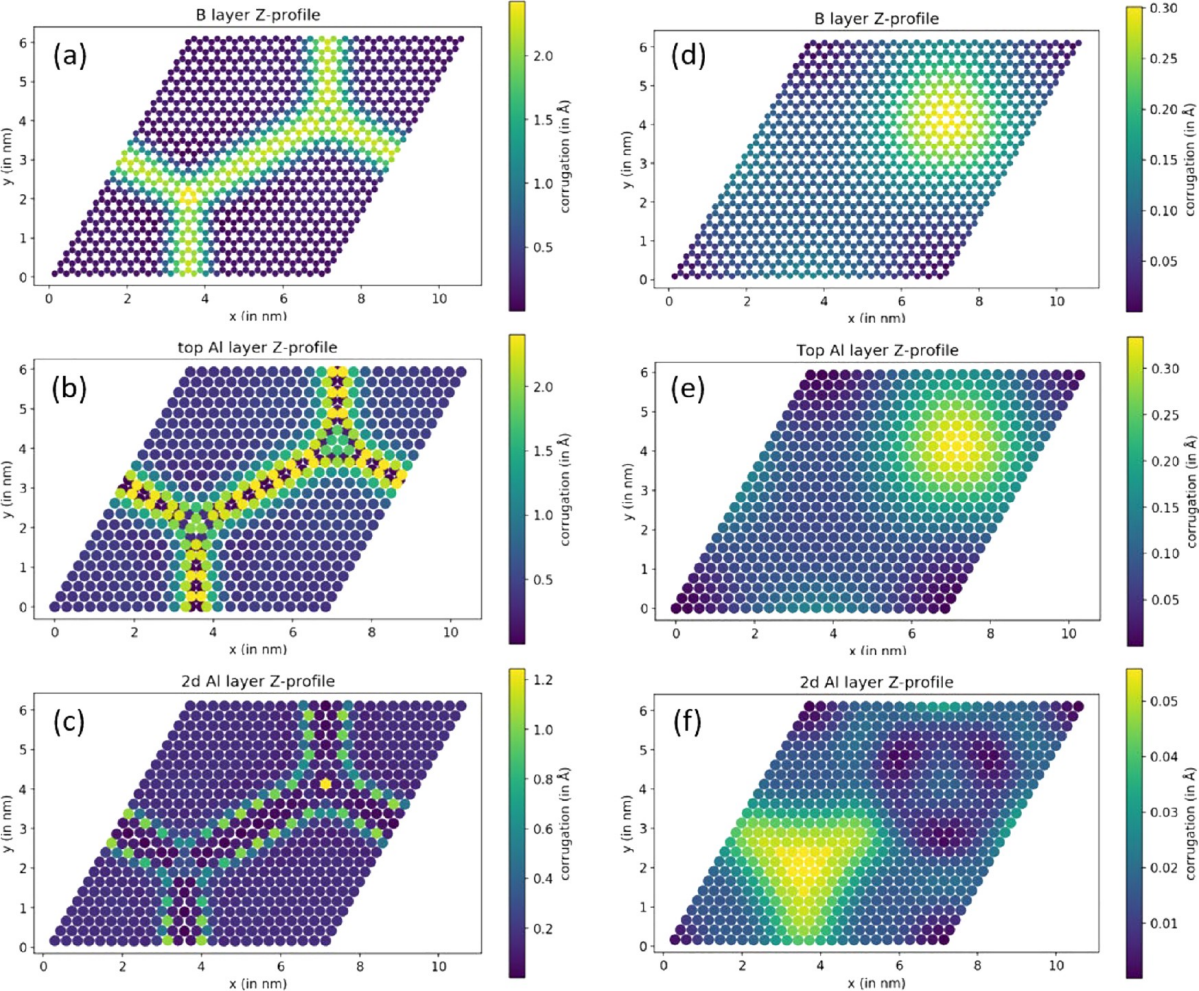
Layer-by-layer height profile maps for two models under study after DFT geometry optimization. For each model the profile map is shown for the honeycomb B layer (model 1 in (a) and model 2 in (d)), the top Al layer (model 1 in (b) and model 2 in (e)), and the second Al layer (model 1 in (c) and model 2 in (f)). Notice the strong difference in corrugation figures between the two models.

A result of the geometry optimization for both models is shown in Figure 3, presenting height profiles for the HB layer and two upper Al layers. In addition, top and side views of the optimized models are shown in Figure S2 of the Supporting Information. As can be seen from these figures, there is a drastic difference in the optimized geometry between models 1 and 2. For model 1, the HB layer and top Al layer become both strongly corrugated in-phase, with the amplitude of corrugation as large as 2.4 Å (Figure 3a,b). This corrugation is a result of the behavior of Al atoms in the top Al layer, which try to become lattice-matched to the HB sheet at as large a portion of the surface as possible. Particularly, the top Al layer expands compared to the underlying layers (Figure 3b) and matches 1:1 in the corners of the supercell (corresponding to the hcp-fcc sites for B adsorption, as shown in Figure 2a). The excessive atoms of the top Al layer are pushed toward the lines connecting top-hcp and top-fcc adsorption sites and pressed down into the second layer, thus elevating the HB very significantly. These atoms are also very disturbing for the second Al layer, resulting in a significant corrugation there too (Figure 3c). Adding one more Al layer and allowing three top Al layers to relax results in preserving a similar level of corrugation amplitude and symmetry, although changing details of the corrugation pattern to some extent (Figure S3).

In model 2 the top Al layer is less dense and readily matched to the HB layer; therefore there are no excess Al atoms to be repelled from this layer. As a result, this model converges to its optimized geometry faster and all layers in the model are disturbed much less than in model 1. The corrugation of the HB layer and the top Al layer is again in-phase (see Figure 3d,e), but its amplitude is not larger than 0.35 Å, being considerably closer to the experimentally measured values. The second Al layer is corrugated in antiphase with the top Al layer, but this corrugation is marginal, around 0.05 Å (Figure 3f). Overall, the formation of a matched AlB_2_ layer on the surface seems to be unavoidable: it actually occurs even starting from model 1. As the high corrugation amplitude and the bright hexagonal pattern with supercell periodicity characteristic for model 1 were never observed in experiment, we conclude that upon growth the excessive Al atoms are pushed away from the areas covered by HB islands, giving space to a weakly corrugated matched AlB_2_ layer, represented by model 2. This conclusion can be further supported by calculating the formation energy (FE) in both systems, defined as
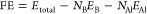
where *E*_B_ is a reference energy of one B atom in the HB and *E*_Al_ is a reference energy of one Al atom in the bulk Al. *N*_B_ and *N*_Al_ are the numbers of B and Al atoms in our systems. For the supercell in the optimized models 1 and 2 the calculated FEs are −704.05 and −782.76 eV, respectively. Therefore, model 2 is considerably more stable than model 1. In the following we will use the terms “HB on Al(111)” and “one-layer AlB_2_” as equivalents.

**Figure 4:**
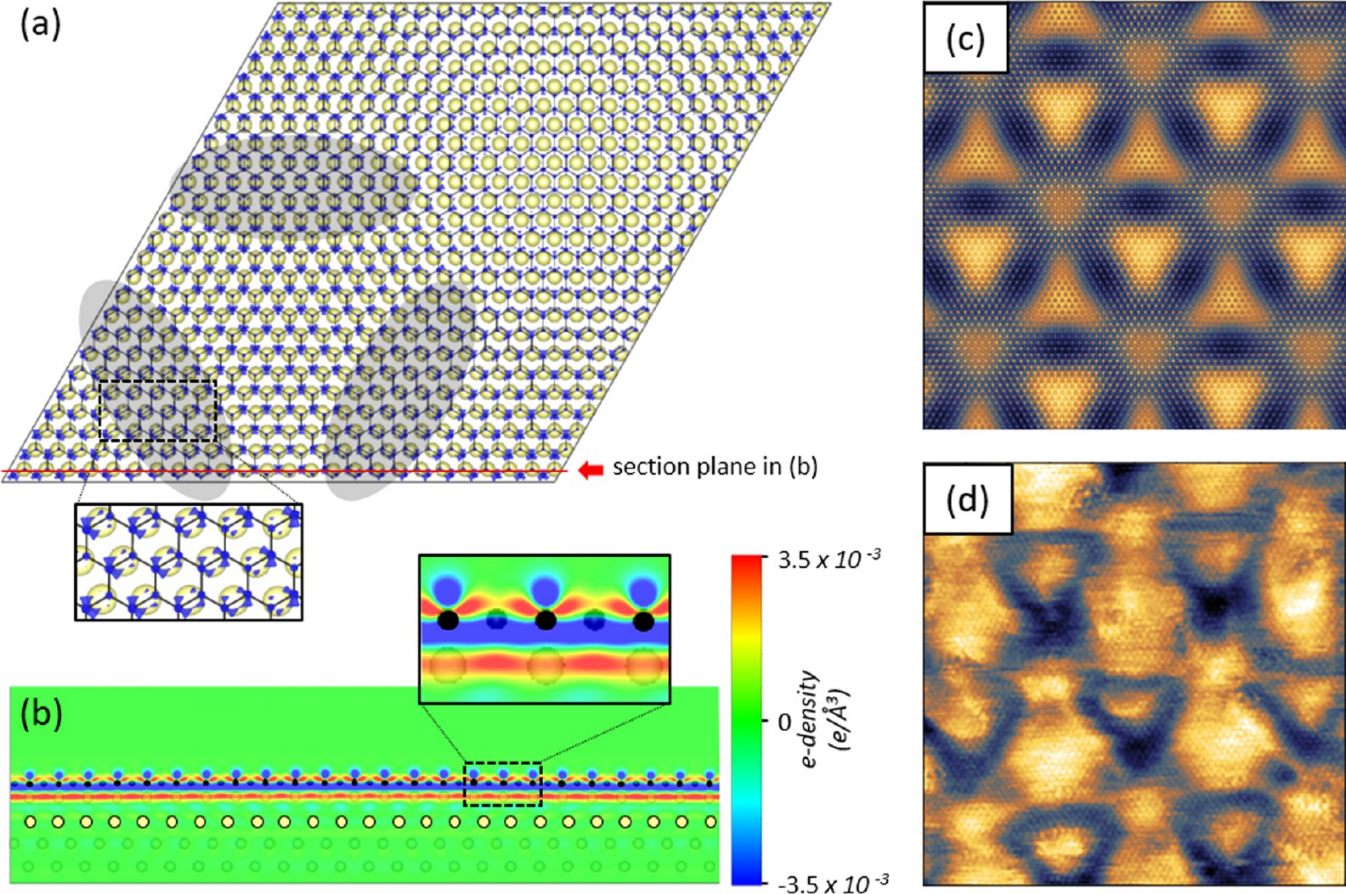
Origin of the supercell contrast in STM for the AlB_2_ monolayer on Al(111). (a) Isosurfaces of differential electron density (blue color, Δρ = −0.007 e/Å^3^) superimposed on the HB mesh (black lines) and second Al layer (light yellow balls). Top Al layer is lattice-matched to HB and is therefore omitted for clarity. Areas where HB is in bridge position relative to the second Al layer are shown in gray; a zoomed-in fragment of this area is shown in the inset. (b) Cross-section of the charge density along the red line in (a). This section is cut through one row of B atoms (black) and one row of Al atoms in the second layer (light yellow); projections of other atoms are shown half-transparent. (c) Simulation of the STM image in the constant current mode (20 × 20 nm, average tip height 4.7 Å above the top B layer). (d) Corresponding 20 × 20 nm STM image.

The experimental STM images of HB on Al(111) in Figure 1 and in the previous works^10,14^ show a peculiar triangular feature in each supercell with a darker contrast on the rim of this triangle. It is interesting to reveal the mechanism behind this contrast, as pure topography (Figure 3d,e) does not reproduce it well. To do so, we have calculated differential electron density Δρ for geometry-optimized model 2:
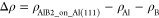
where the latter two electron densities are calculated at the positions of Al and B atoms in the geometry-optimized model 2 (*i*.*e*., single AlB_2_ layer on Al(111)), and we performed simulations of the STM patterns based on this distribution. In Figure 4a an example of a Δρ isosurface is shown, superimposed on the HB mesh and the second Al layer. The top Al layer is 1:1 lattice-matched to HB, forming a layer of AlB_2_; it is not contributing to the contrast across the supercell more than the HB layer and therefore omitted here for clarity. An example of in-depth cross section of Δρ in Figure 4b provides evidence for the formation of a strong dipole in the top AlB_2_ layer. From our Bader charge analysis we conclude that in this dipole layer the differential negative charge per B atom is 0.716*e*, while the differential positive charge per Al atom is 1.448*e*, thus making the remaining Al layers in the slab nearly electron-neutral. Small variations of electron density in this dipole layer across the supercell are due to the mismatch to the underlying Al substrate and must be causing the observed STM contrast. Indeed, as can be seen from Figure 4a, in the areas outlined by gray ovals the negative electron density is spread to some degree along the B–B bonds, while in other areas it is mainly concentrated on the B atoms. We assume that these areas are responsible for the formation of darker rims around brighter triangles, as can be seen in the STM simulation shown in Figure 4c. This simulation provides a reasonably good match to the experimental STM images (Figure 4d). Therefore, we believe that the STM contrast in this system originates essentially from the variations of electron density of the surface dipole film across the supercell rather than by real topography.

**Figure 5:**
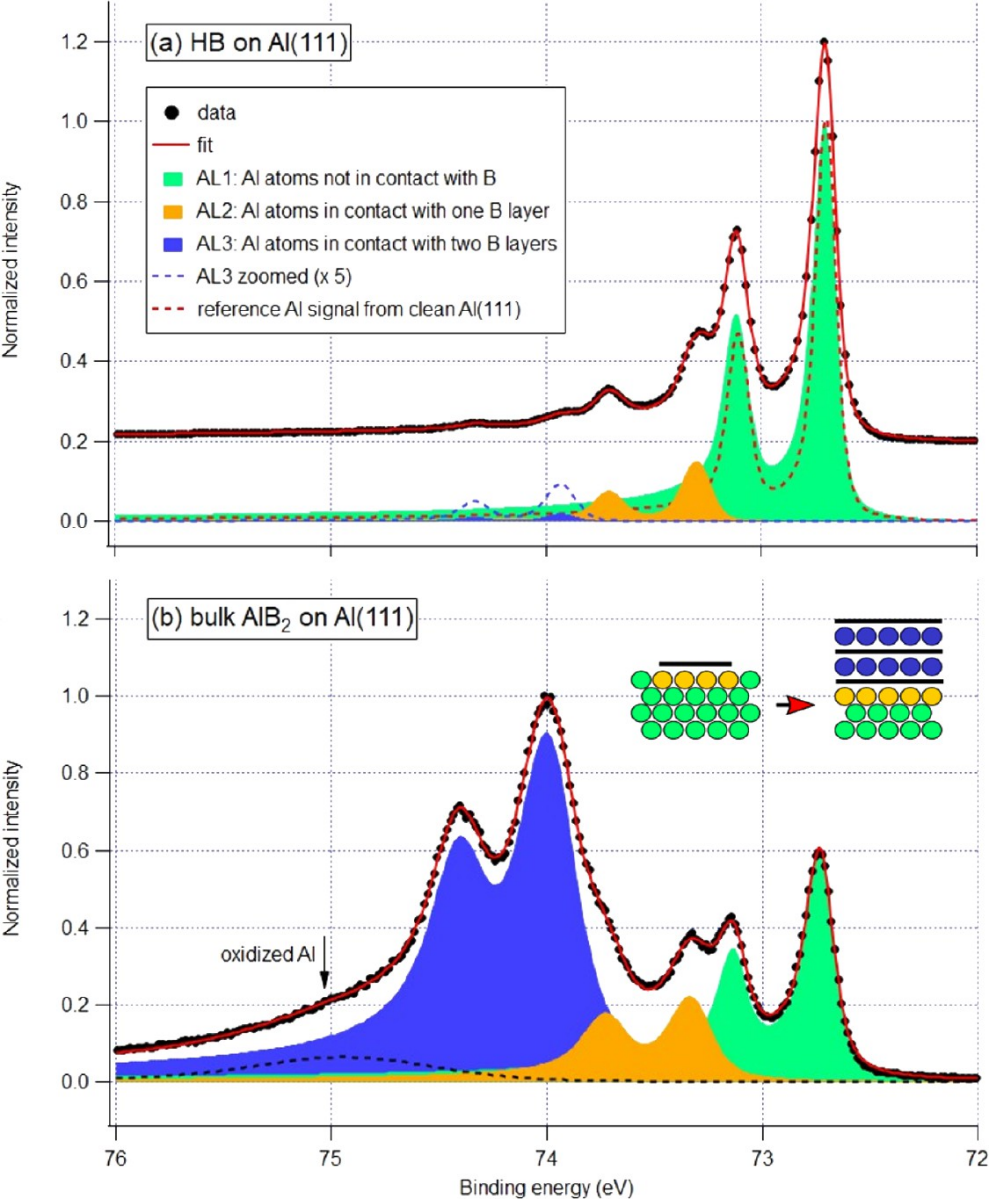
Al 2p XPS spectra. (a) HB on Al(111), *ca*. 0.5 ML in terms of HB, *h*ν = 150 eV. (b) AlB_2_ on Al(111), *ca*. 3 ML in terms of HB, *h*ν = 450 eV. A small signature of Al oxidation is visible at 75 eV. A schematic in the center shows a transformation from (a) to (b), where balls represent Al atoms and black lines represent layers of honeycomb boron; each ball color reflects the color of a corresponding spin–orbit doublet in the spectra.

The theoretically predicted large electron transfer from Al to B is crucial for stabilizing HB on Al(111).^10,15^ In order to provide experimental evidence for this charge transfer, we have studied the Al 2p XPS spectra from clean Al(111), HB on Al(111), and bulk AlB_2_ on Al(111). Typical high-resolution Al 2p spectra are shown in Figure 5 on the binding energy (BE) scale, as referred to the Fermi level. The spectra from samples with no or low B coverage are taken with photon energy *h*ν = 150 eV for higher surface sensitivity, while the spectrum from a sample with large coverage is taken with *h*ν = 450 eV for better visualization of both bulk and interface signals.

The spectrum from clean Al(111) (red dotted line in Figure 5a) shows a single spin–orbit doublet with the Al 2p_3/2_ BE of 72.70 eV and spin–orbit splitting of 0.40 eV, indicating that Al atoms of the top layer are in the same chemical state as the bulk atoms despite the lack of coordination. Once a layer of HB begins to form on Al(111), a second spin–orbit doublet at 73.30 eV starts to grow in the Al 2p spectrum (AL2 in Figure 5) accompanied by a low-intensity third doublet (AL3) at 73.93 eV. We assign the component denoted AL2 to the manifestation of interfacial interaction between the top layer of Al and HB; the high-BE shift of 0.6 eV reflects a strong electron donation from this layer to HB. The AL3 doublet is plausible to associate with the nucleation of two-layer AlB_2_ islands observed in STM (Figure 1a), because the Al atoms associated with it are embedded between two layers of honeycomb boron, thus being more depleted of electrons than Al atoms with B neighbors on one side only. Interestingly, the high-BE shift of AL3 from the “neutral” AL1 component is 1.2 eV, twice as large as for the AL2 component. This interpretation gains further support upon studying the Al 2p spectrum from a sample with high B coverage (nominally three or four layers of AlB_2_) shown in Figure 5b. Here we see the same three doublets, AL1, AL2 and AL3, at nearly the same energy, but the dominating one now is AL3, as a clear evidence of massive formation of bulk AlB_2_ on Al(111). It is obvious from Figure 5 that the chemical state of Al atoms in contact with only one honeycomb boron layer (either HB on top of Al(111) or the layer at the interface between Al(111) and AlB_2_) is very different from that in bulk AlB_2_.

**Figure 6:**
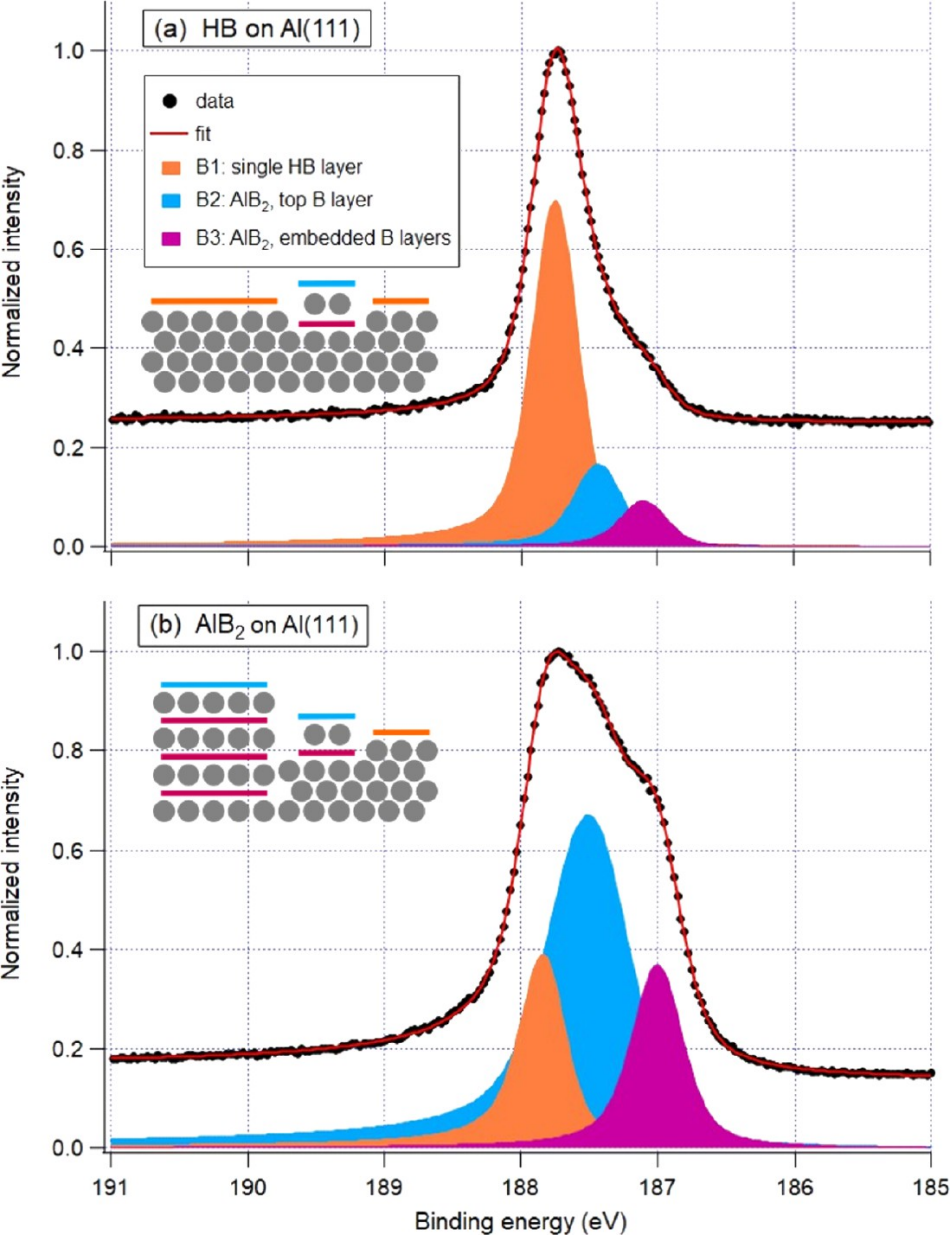
B 1s XPS spectra. (a) HB on Al(111), *ca*. 0.5 ML in terms of HB, *h*ν = 270 eV. (b) AlB_2_ on Al(111), *ca*. 3 ML in terms of HB, *h*ν = 270 eV. In the schematics, gray balls represent Al atoms and colored lines represent layers of honeycomb boron; each line color reflects the color of a corresponding component in the spectra.

Further information on the structure and electronic properties of HB and AlB_2_ can be gained from the B 1s XPS spectra. In borophene on Ag(111), B atoms can be coordinated by four, five, or six B neighbors, and the B 1s BE was reported to vary for boron atoms with different coordination numbers.^28^ Different from Ag(111), on Al(111) all B atoms are coordinated by only three B neighbors; therefore only one component is expected in the first approximation in the B 1s XPS spectrum of HB on Al(111). Indeed, as can be seen from Figure 6a, this spectrum is dominated by one strong component, B1 (colored orange), at the BE of 187.75 eV. However, it is accompanied by two smaller components, B2 (light blue) and B3 (magenta), at the BE of 187.44 and 187.08 eV, respectively. To identify these components, we varied the electron emission angle and observed that B2 is gradually increased in intensity relative to both B1 and B3 upon going away from normal emission (as shown in Figure S4 in the Supporting Information). This is a clear indication of B atoms being elevated relative to the standard HB layer. Therefore, B2 originates from the honeycomb boron layer on top of the AlB_2_ islands, while B3 must be due to the embedded boron layer, as illustrated in the schematics in Figure 6a. The intensity of B2 is around 20% of the total B1 + B2 intensity from all on-top boron atoms, which is in agreement with STM observations mentioned earlier. (The fraction of the area occupied by two-layer AlB_2_ islands in the total HB coverage can vary to some degree from one preparation to another.) With increasing B coverage and the thickness of the AlB_2_ film the intensities of both B2 and B3 are increasing, while B1 becomes less and less pronounced (Figure 6b), thus confirming the above assignment.

Up to now we demonstrated that XPS results are fully consistent with STM and LEED, allowing us to monitor and control the formation of HB (*i.e*., one-layer AlB_2_) and then bulk AlB_2_ on Al(111) by observing their peculiar spectroscopic signatures. Interestingly, a rather small width of the B1 component in B 1s XPS from HB (0.4 eV, see Figure 6a) indicates that all sp^2^-bonded B atoms are in a close chemical state on Al(111). In that sense HB on Al(111) is reminiscent of graphene, but the strong chemical B–Al interaction causing the formation of a stoichiometric one-layer AlB_2_ may affect or even destroy pronounced spatial and energy separation of the π- and σ-electron subsystems characteristic for the latter. Therefore, it is interesting to reveal to what extent the HB is actually similar to graphene.

**Figure 7:**
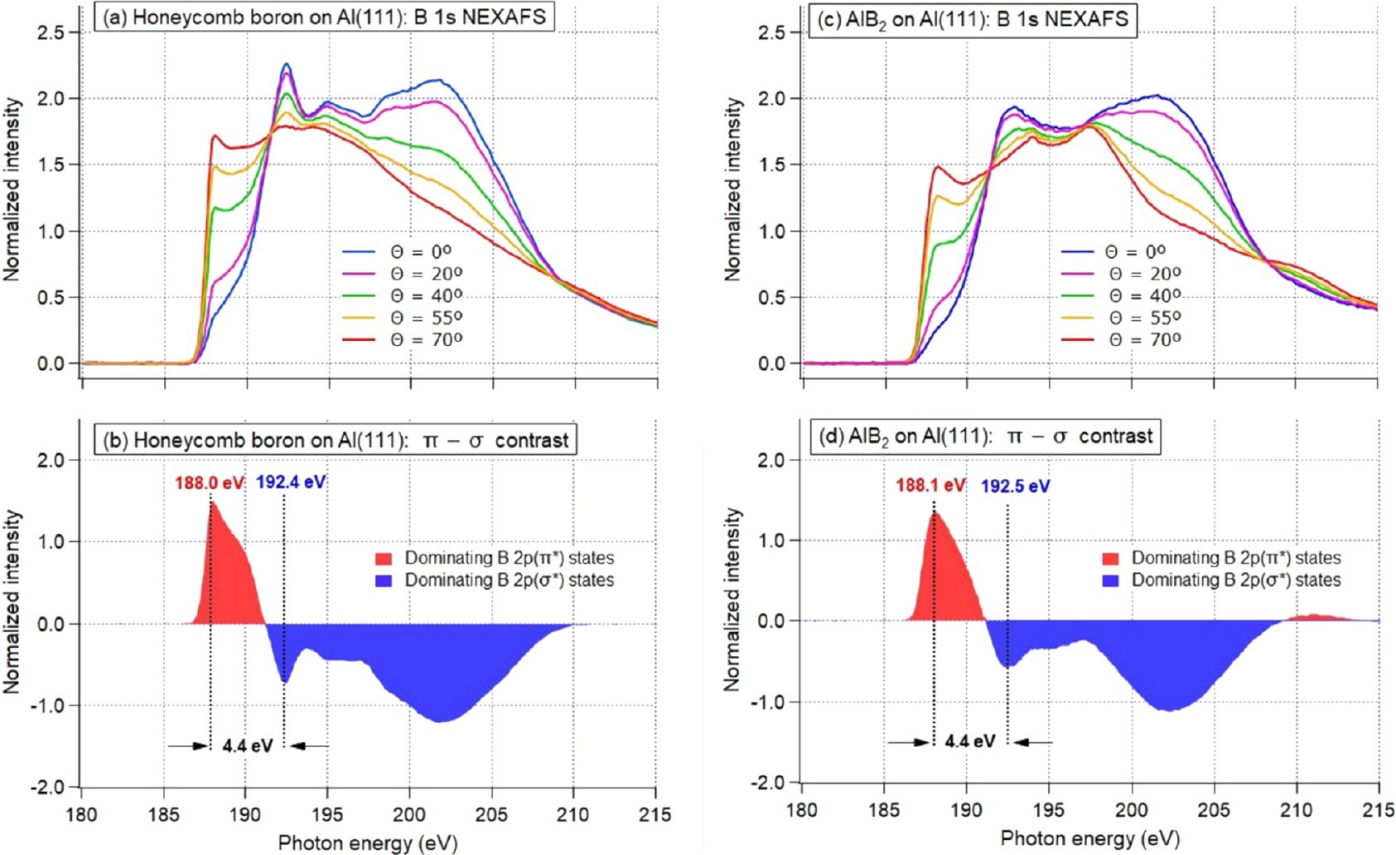
B 1s NEXAFS spectra as a function of angle Θ between the surface plane and the direction of photon polarization from (a) HB on Al(111), *ca*. 0.5 ML in terms of HB, and (c) AlB_2_ on Al(111), *ca*. 3 ML in terms of HB. (b and d) Difference curves for spectra taken at 70° and 0° for (a) and (c), respectively, with an additional geometry correction applied to approximate the pure π* – σ* difference (as a spectrum at 90° cannot be measured).

Angle-dependent NEXAFS spectroscopy is known as an ideal tool for probing π* and σ* states in 2D materials, with a textbook example of graphite^29^ or more recent examples of monolayer hexagonal boron nitride^30^ or graphene^31^ on metal surfaces. In order to directly probe symmetry and composition of the HB electronic system, we studied angle-dependent B 1s NEXAFS spectra from submonolayer HB (Figure 7a) and few-layer AlB_2_ (Figure 7c) grown on Al(111). All NEXAFS spectra in Figure 7 are normalized to the incident photon intensity and to the integral intensity under the curves. No sign of boron oxidation due to contaminations is visible in the spectra apart from a tiny peak appearing at grazing angles at 194.0 eV in the spectra of AlB_2_ (Figure 7c). This is a B 1s → 2p(π*) resonance in the B 1s NEXAFS spectrum of the BO_3_ group;^32^ from its intensity the oxidation can be considered negligible. The spectra from HB (Figure 7a) demonstrate strong and clear angle dependence, as visualized further by a π* – σ* difference curve in Figure 7b. Spectral intensity below 191.3 eV reflects transitions from B 1s to the unoccupied electronic states with predominantly π (out-of-plane) character with a pronounced B 1s → 2p(π*) resonance at 188.0 eV. Above 191.3 eV the spectra are dominated by transitions to σ-like (in-plane) states, and the first sharp B 1s → 2p(σ*) resonance is seen at 192.4 eV. Therefore, an energy separation between the π* and the σ* resonances, Δ*E*(π*−σ*), is equal to 4.4 eV in HB on Al(111). The NEXAFS spectra (Figure 7c) and the π* – σ* difference curve (Figure 7d) from a few-layer (bulk) AlB_2_ film are very similar to those from HB on Al(111) (Figure 7a and b), apart from less sharp features due to reduced sample ordering and a bulk-related structure appearing around 210 eV. The spectral shape is determined not purely by the sp^2^-hybridization on B atoms but also by the interaction of HB with Al. Therefore, the observed similarity of the B 1s NEXAFS spectra from HB on Al(111) and from AlB_2_ indicates that this interaction is the same in both cases, thus providing direct experimental evidence that HB is forming a stoichiometric one-layer AlB_2_ on top of Al(111)..

The position of the first absorption π* resonance E (B 1s → 2p(π*)) and the energy separation Δ*E*(π*−σ*) are important parameters of the B 1s NEXAFS. It can be demonstrated that their values (188.0 and 4.4 eV, respectively) provide direct spectroscopic evidence for the fact that B atoms are in the sp^2^-bonded configuration on Al(111). Indeed, let us compare these two parameters in the B 1s NEXAFS spectra of compounds where the B atom is surrounded in the same planar triangular geometry (*D*_3*h*_ point symmetry group) by atoms with gradually decreasing electronegativity (see Table 1).

**Table 1:** Position of the First Absorption π* Resonance and π*−σ* Energy Separation in the B 1s NEXAFS Spectra of Planar Triangular BX_3_ Species (X = F, O, Cl, N, Br, B Atoms)

compound/molecule	formal boron surrounding	Pauling electronegativity	*E*(B 1s → 2p(π*)), eV	Δ*E*(π*−σ*), eV	bond length, pm	NEXAFS reference
BF_3_	**B-**F_3_	F: 3.98	195.9	10.1	130	(33)
H_3_BO_3_, B_2_O_3_	**B-**O_3_	O: 3.44	193.95	8.5	136	(32)
BCl_3_	**B**-Cl_3_	Cl: 3.16	192.4	4.5	174	(34)
h-BN	**B-**N_3_	N: 3.04	191.8	6.8	145	(35)
BBr_3_	**B-**Br_3_	Br: 2.96	192.0	3.8	190	(34)
HB on Al(111)	**B-**B_3_	B: 2.04	188.0	4.4	172 (exptl), 171.5 (calcd)	this work

**Figure 8:**
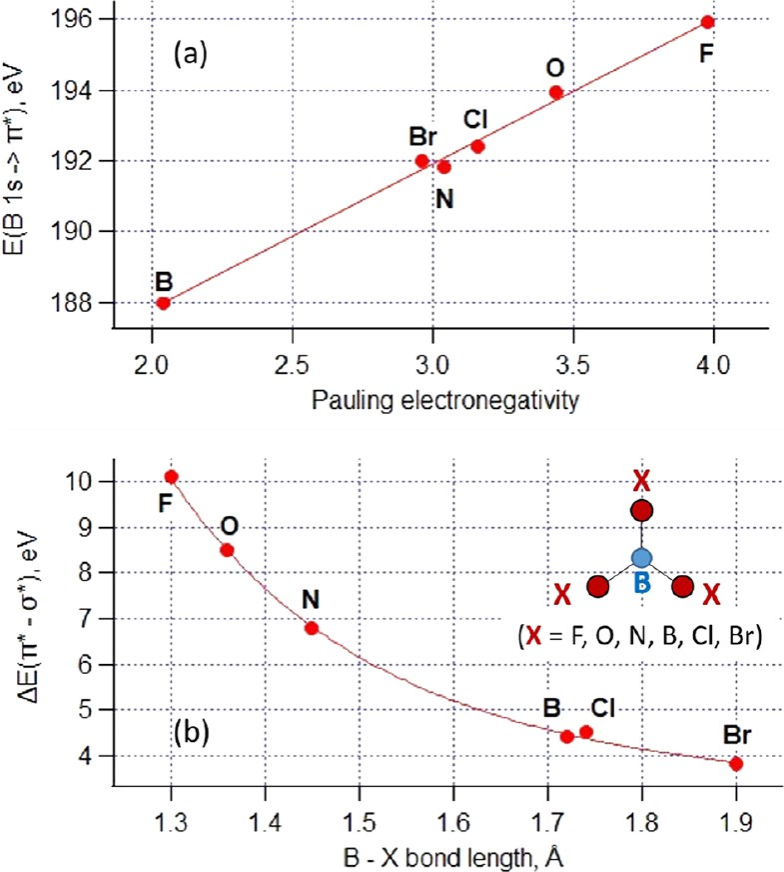
Chemical trends in the B 1s NEXAFS spectra of planar triangular BX_3_ (X = F, O, N, B, Cl, Br) groups. (a) Energy of the B 1s → 2p(π*) transition as a function of Pauling electronegativity of the B surrounding; (b) π*−σ* energy separation as a function of the B–X bond length.

As the neighbors of the central B atom are varying in the sequence B → Br → N → Cl → O → F, the position of the B 1s → π* resonance is gradually shifted to higher energy (Figure 8a). Similar to the initial-state effect in XPS, this shift is mainly due to the deepening of the B 1s core level upon depleting electron density on the absorbing B atom, thus reflecting a gradual increase in the bond ionicity. Another interesting trend is the dependence of energy separation Δ*E*(π*−σ*) on the bond length. As can be seen from Figure 8b, it is reduced monotonously upon weakening and elongation of the B–X bond in the BX_3_ planar triangular group (X = F, O, N, B, Cl, Br). This effect is rather common in linear and planar molecules and quasi-molecular groups:^36^ the stronger (shorter) the bonds, the stronger the anisotropy of the molecular potential around the absorbing atom, resulting in larger energy separation between states of 2pπ and 2pσ symmetry. The case of HB on Al(111) is a good match to both trends in Figure 8, thus providing evidence for a truly two-dimensional honeycomb material despite a substantial interaction with Al and formation of a stoichiometric one-layer AlB_2_.

**Figure 9:**
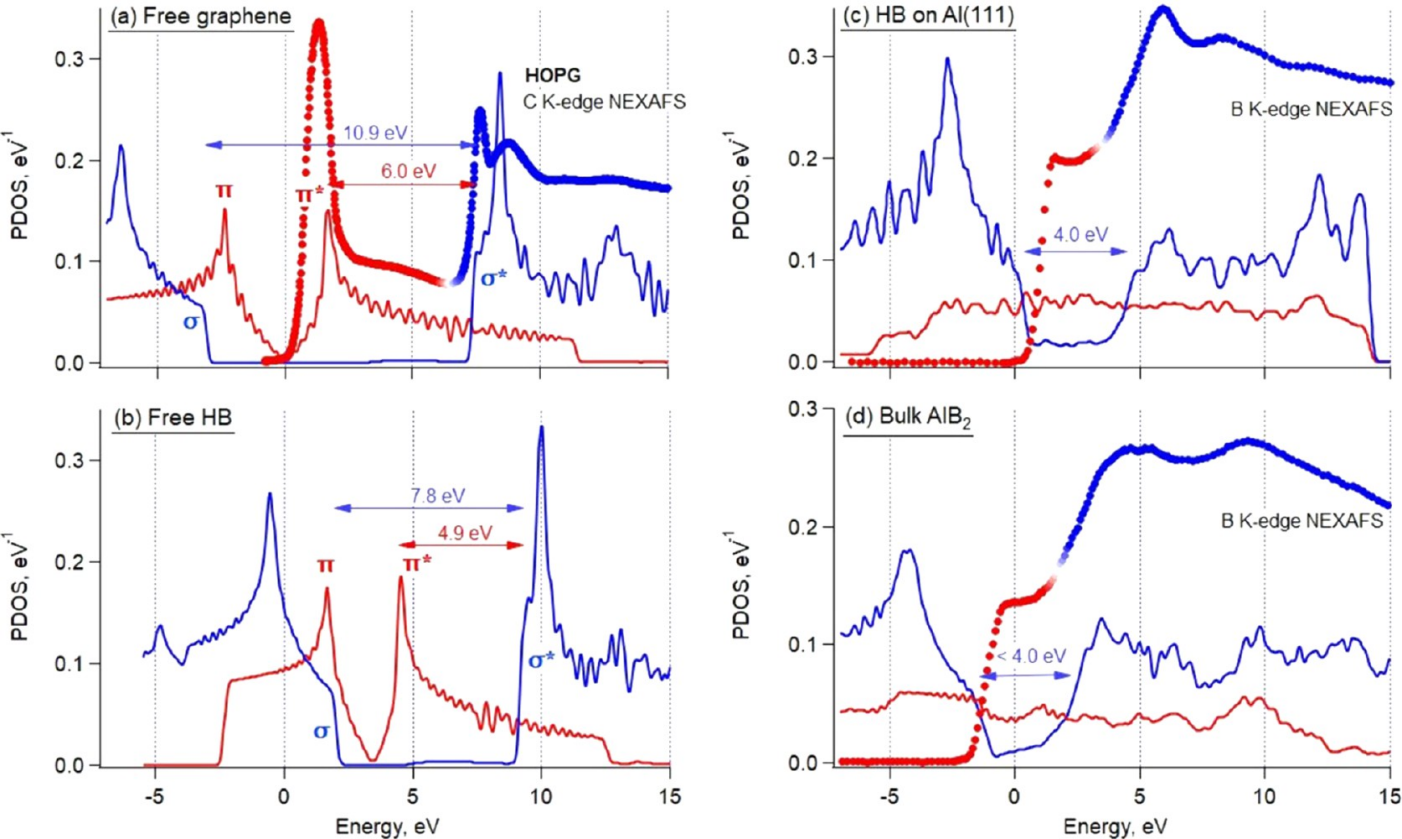
Calculated partial DOS per atom compared with NEXAFS spectra. 2p_*z*_ (out-of-plane) PDOS curves are shown in red, 2s+2p_*x*_+2p_*y*_ (in-plane) PDOS curves are blue; energy scale is relative to the Fermi level. NEXAFS spectra are aligned to the DOS curves at the position of the first σ* resonance and are shown in colored dots to reflect transitions to the π* (red) and σ* (blue) states. Energy separations Δ*E*(π* – σ*) and Δ*E*(σ – σ*) are indicated by red and blue arrows with numbers, respectively. (a) C 2p PDOS in freestanding graphene and C 1s NEXAFS from HOPG; (b) B 2p PDOS in freestanding HB; (c) B 2p PDOS and B 1s NEXAFS from HB on Al(111); (d) B 2p PDOS in bulk AlB_2_ and B 1s NEXAFS from a few-layer AlB_2_ film.

It is very instructive to compare NEXAFS spectra with the theoretical calculations of partial density of states (PDOS) in graphene, HB, and bulk AlB_2_. Apparently, DOS curves cannot reproduce the NEXAFS shape completely, because the transition probabilities and core–hole influence are not considered, but it can often help to understand spectra qualitatively. In the case of 2D materials, PDOS calculations are particularly helpful for comparison with NEXAFS because they demonstrate clear energy separation between π- and σ-electronic subsystems. For example, such a separation between 2p_*z*_ (π) and 2s,p_*x*,*y*_ (σ) states is clearly visible in the C 2s,p PDOS for freestanding graphene, giving a very good match to the C 1s NEXAFS spectrum of highly oriented pyrolytic graphite (HOPG) in Figure 9a. On going from freestanding graphene to freestanding HB (Figure 9b), the overall structure of the PDOS remains the same (and very similar to the previously reported calculations^2,15^) with two essential distinctions from the case of graphene. First, the σ–σ* and π*−σ* energy separations are reduced because of bond elongation in the sp^2^-bonded boron as compared to graphene (172 pm *vs* 142 pm), in agreement with the trend discussed above and shown in Figure 8b. Second, the position of the Dirac point (crossing between π and π* states) has shifted from 0 relative to the Fermi level in graphene to 3.5 eV in free-HB. This shift is a signature of electron deficiency in the bonding states of free-HB responsible for its instability, as explained in the previous theoretical works.^2,15^

The B 2s,p PDOS in HB on Al(111) after supercell geometry optimization of model 2 (as in Figure 3d) are shown in Figure 9c. These curves from supercell calculations can be compared with the DOS curves computed in the approximation of a (1 × 1) matching between B and Al lattices and B atoms placed at fcc and hcp hollow sites on Al(111).^15^ (We have also reproduced the latter calculations in the Supporting Information to facilitate the comparison; see Figure S5.) Although minor details differ in different calculations for HB on Al(111), it is clear that the B 2p(π) states are greatly affected by mixing with the Al 3s,p states, while the B 2p(σ) states are influenced less. Still, although a well-defined σ – σ* energy separation yet exists on going from freestanding to Al-supported HB, it is reduced from 7.8 eV to around 4.0 eV, reflecting a broadening of the σ bands upon placing HB on Al(111). As for the π* states, they are smeared out into a broad band, as confirmed by the B 1s NEXAFS spectrum in Figure 9c. The calculated DOS can describe the experimental B 1s NEXAFS spectra from Figure 7 quite accurately: the π* resonance broadened into a continuum of some 4 eV followed by a pronounced σ* resonance. The supercell calculation cannot completely reproduce an absence of σ states at the Fermi level, even though it does better in this respect than the “lattice-matched” calculation (see Figure S5 in the Supporting Information). Notice that in the PDOS calculation for bulk AlB_2_ (Figure 9d) this problem is gone: there are no B 2p(σ) states at the Fermi level, as it is expected from the NEXAFS spectra and from the ARPES data for bulk AlB_2_.^37^ We cannot say for sure whether some B 2p(σ) states are actually present at the very Fermi edge for HB on Al(111); the fact that we do not observe these states in the NEXAFS spectra may be due to the influence of the core hole.

For comparison, it would be interesting to assess chemical interaction between graphene and Al(111). Although a direct growth of epitaxial graphene on this surface is hardly possible by CVD due to the lack of catalytic activity, it is possible to form graphene on a more active substrate and then intercalate an Al monolayer in-between. For the case of graphene on Ni(111) it has been shown that such intercalation of Al effectively decouples graphene from the substrate, and the C 1s NEXAFS spectra clearly show a quasi-freestanding nature of graphene on Al/Ni(111).^31^ Therefore, the chemical interaction at the graphene/Al interface is probably weak, in difference from that at the HB/Al interface.

All in all, we suggest that “honeycomb borophene” in the form of a graphene-like quasi-freestanding 2D layer can hardly exist on Al(111). Instead, due to strong Al–B interaction and ease of reconstructing the Al substrate, the topmost honeycomb boron is an integral part of the surface AlB_2_ layer. Despite strong chemical interaction with the substrate, the electronic structure of honeycomb boron on Al(111) preserves certain similarity to that of hypothetical freestanding honeycomb boron, as can be judged from the comparison of NEXAFS data and DFT calculations.

## Conclusions

The interface between honeycomb boron and Al(111) has been studied by means of core-level synchrotron-based spectroscopies, STM, LEED, and large-scale DFT calculations in an attempt to reveal exact atomic arrangement, peculiarities of the growth process, and details of the electronic structure.

From DFT calculations of the 24 × 25 superstructure we conclude that HB can grow on Al(111), but not as a quasi-freestanding monolayer. Instead, it is intimately bound and lattice-matched to the top metal layer, forming a stoichiometric one-layer AlB_2_, which is in turn lattice-matched in the ratio 24:25 to the underlying Al substrate. This finding is supported experimentally by close similarity between the angle-resolved B 1s NEXAFS spectra from HB on Al(111) and few-layer AlB_2_ on Al(111) and is in agreement with the recent ARPES study by Geng *etal*.^14^ In this respect, the term “honeycomb borophene” may not be very appropriate for this system, because the B layer can hardly become freestanding, different from graphene or monolayer h-BN on lattice-mismatched metal surfaces. This difference is due to strong chemical bonding between HB and the supporting Al layer. On the other hand, sp^2^-hybridized B 2s,p states can still be identified in the one-layer and few-layer AlB_2_ by NEXAFS spectroscopy, despite the strong B–Al interaction, indicating certain similarity with graphene. The strong and clear energy splitting between B 2p(π*) and B 2s,p(σ*) states in the B 1s NEXAFS spectra reflects an essentially 2D nature of the electron subsystem in both one-layer AlB_2_ and bulk AlB_2_.

The top AlB_2_ forms a strong dipole on the surface, with a massive negative charge on the topmost boron layer (−0.716*e* per B atom) and equally strong positive charge on the underlying Al layer (+1.448*e* per Al atom), leaving the rest of the Al atoms nearly neutral. The triangular-shaped superstructures observed in the STM images from HB on Al(111) are due to the mismatch between the top AlB_2_ layer and the second Al layer and can be reproduced by theoretical STM simulations fairly well.

With increasing B coverage, thicker AlB_2_ films are formed in the course of intercalation of B atoms under already formed AlB_2_ layers and embedding into the bulk of an Al crystal. For these thicker films, the triangular-shaped superstructure in STM is fading away, as several AlB_2_ layers become stacked in a lattice-matched fashion on top of each other.

## Methods

### Experiments

All experiments were performed at the MAX IV laboratory in Lund, Sweden. For the STM/LEED studies, we used a two-chamber ultra-high-vacuum (UHV) system, featuring a boron evaporator (EFM 3 from Focus GmbH) and a LEED spectrometer (OCI Vacuum Microengineering Inc.) in the preparation chamber and a variable-temperature STM (VT XA SPM from Scienta-Omicron) in the analysis chamber, with the overall base pressure better than 1 × 10^–10^ mbar. All LEED and STM measurements were performed at room temperature. All bias voltages for the latter are defined as the tip bias with respect to the grounded sample. Values between −2 and 2 V were used for imaging; we did not observe any significant impact of the bias voltage on the apparent height of the boron-induced islands. Gwyddion software^17^ was used for STM image processing.

Spectroscopic studies were carried out at the Surface and Material Science branch of the FlexPES beamline using a SES-2002 photoelectron analyzer (Scienta-Omicron) for XPS and a home-built partial electron yield multichannel-plate detector for recording NEXAFS spectra (with retardation voltage set to −140 V for the B 1s NEXAFS spectra). A similar LEED spectrometer (also from OCI Vacuum Microengineering Inc.) was used in the spectroscopy end station to crosscheck sample quality. The overall energy resolution in XPS was set to 25 meV (Al 2p, photon energy 150 eV) and 75 meV (B 1s, photon energy 270 eV); in the B 1s NEXAFS spectra it was set to 15 meV. The base pressure did not exceed 3 × 10^–10^ mbar. Samples were grown *in situ* in both locations with similar experimental conditions; several samples were transferred from one location to the other in a vacuum suitcase (base pressure better than 5 × 10^–10^ mbar) to double-check reproducibility of results.

Single-crystal Al(111) was cleaned by repeated cycles of soft Ar^+^ sputtering (<400 eV) followed by annealing to approximately 550 °C. Boron was evaporated from the pure boron rod (99.999%) of ∼5 mm diameter by electron beam heating. The sample temperature was controlled by a thermocouple directly attached to the sample. For a satisfactory growth quality, the sample temperature could vary in the range from 150 to 250 °C, with the best quality of HB obtained around 180 °C. The pressure during deposition was better than 5 × 10^–10^ mbar. In total more than 25 samples with different B coverage (from 0.5 to 3 ML in terms of HB layers) and growth temperature were studied.

### Calculations

DFT calculations were performed using the generalized gradient-corrected approximation with the parametrization of Perdew–Burke–Ernzerhof (PBE)^18^ for the exchange–correlation functional and the projector-augmented wave (PAW) formalism as implemented in the Vienna ab initio simulation package (VASP)^19,20^ if not stated otherwise. A plane wave basis set with an energy cutoff of 400 eV was used. The face-centered cubic (fcc) lattice of Al was optimized using the Monkhorst–Pack 24 × 24 × 24 *k*-point mesh for Brillouin zone sampling. The calculated Al lattice parameter *a* = 4.0398 Å is in excellent agreement with its experimental value of 4.04958 Å.^21^ The optimized lattice of bulk Al was used to construct a four-layer 25 × 25 slab of Al(111) surface (2500 Al atoms) with the lattice parameter of *a* = 71.4139 Å. The periodically replicated slabs were separated by a vacuum region of ∼15 Å. In the case of the surface slab, the reciprocal space was spanned using the Γ point due to the large size of the supercell. The free-standing HB structure has been optimized using the Monkhorst–Pack 24 × 24 × 1 *k*-point mesh. The calculated value of the lattice constant of a free-standing HB is 2.9177 Å. For the first model, the 24 × 24 superstructure of the HB sheet (1152 B atoms) was optimized on the 25 × 25 slab of the Al(111) surface until forces were less than 0.01 eV Å^–1^. Upon optimization, only positions of Al atoms in the bottom two layers of the slab were fixed, while all B atoms and Al atoms in the two outermost layers of the slab were allowed to relax. The same procedure was used for the second model, with the only difference that the top Al layer contained fewer atoms (576 instead of 625) being lattice-matched to the HB sheet. The atoms in molecules (AIM) method of Bader was used for charge analysis.^22,23^ The simulated STM image was obtained in the constant current mode based on calculated electron densities using the Tersoff–Hamann model^24,25^ in conjunction with Bardeen’s approximation for tunneling matrix elements.^26^

## Supporting Information Available

Additional STM image showing gradual disappearance of the moiré pattern with AlB_2_ film thickness, top and side views of studied theoretical models before and after geometry optimization, a comparison of the Z-profile maps for model 1 optimized with 4 and 5 Al layers included in the slab, analysis of the B 1s XPS as a function of the electron emission angle, and comparison of the B 2s,p PDOS calculated in lattice-matched approximation and for the 24:25 supercell model (PDF)
