# Supplementary material for: Honeycomb Boron on Al(111): From the Concept of Borophene to the Two-Dimensional Boride
Source: ACS Nano. 2021 Aug 30;15(9):15153–65. doi: 10.1021/acsnano.1c05603 (PMC8482755; doi:10.1021/acsnano.1c05603)
Supplement: Supplementary file 1 — nn1c05603_si_001.pdf [file nn1c05603_si_001.pdf]

## Supporting Information

# *Honeycomb Boron on Al(111): from the Concept of Borophene to the Two-Dimensional Boride*

*Alexei B. Preobrajenski*[†,*], *Andrey Lyalin*[‡,‖], *Tetsuya Taketsugu*[‡,⊤], *Nikolay A. Vinogradov*[†], *Alexander S. Vinogradov*[╜]

[†] MAX IV Laboratory, Lund University, 22100 Lund, Sweden

[‡] Institute for Chemical Reaction Design and Discovery (WPI-ICReDD), Hokkaido University, Kita 21 Nishi 10, Sapporo 001-0021, Japan

[‖] Center for Green Research on Energy and Environmental Materials (GREEN), National Institute for Materials Science, Namiki 1-1, Tsukuba 305-0044, Japan

[⊤] Department of Chemistry, Faculty of Science, Hokkaido University, Kita 10 Nishi 8, Sapporo 060-0810, Japan

[╜] St. Petersburg State University, St. Petersburg, 198504, Russia

*Corresponding author. E-mail: alexei.preobrajenski@maxiv.lu.se

To complement Figure 1 of the paper, the morphology of a few-layer $AlB_2$ film on Al(111) is shown in Figure S1 on a larger scale. One can see that the original moiré pattern with the period of 7.15 nm characteristic for HB/Al(111) is still visible in this image but it is gradually fading away with increasing thickness of the $AlB_2$ film: area “A” -> area “B” -> area “C”, etc. This behavior is in good agreement with the LEED data of Figure 1 showing disappearance of the superstructure diffraction spots with increasing thickness of $AlB_2$.

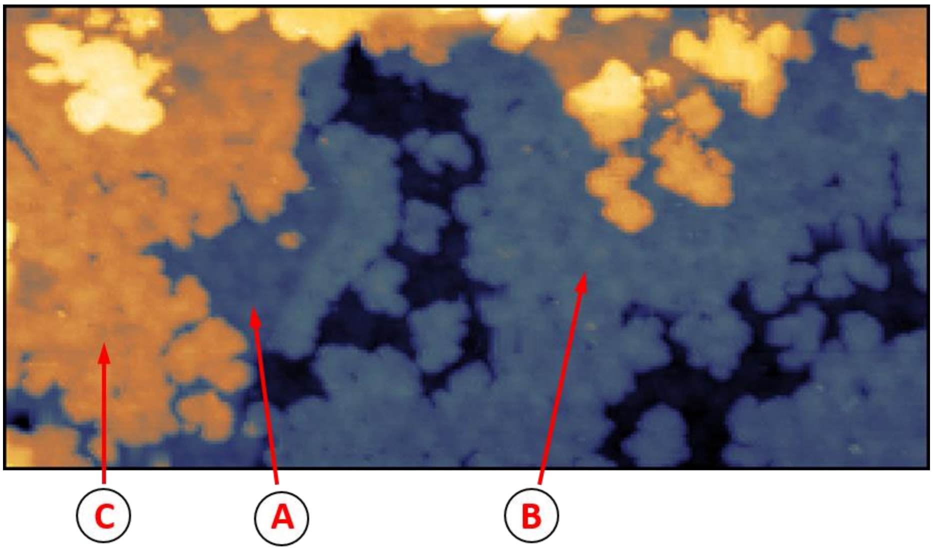


***Figure S1***: *70(h)x35(v) nm STM image recorded from a few-layer $AlB_2$ film grown on Al(111) (scanning parameters are -1.0 V / 280 pA). Areas designated “A”, “B” and “C” correspond approximately to 2, 3 and 4 layers of $AlB_2$, respectively.*

In Figure S2 we show a graphical representation of models 1 and 2 after geometry optimization. Apparently, the disturbance of both Al and B atoms is much more prominent in the case of model 1 (c) than in the case of model 2 (d).

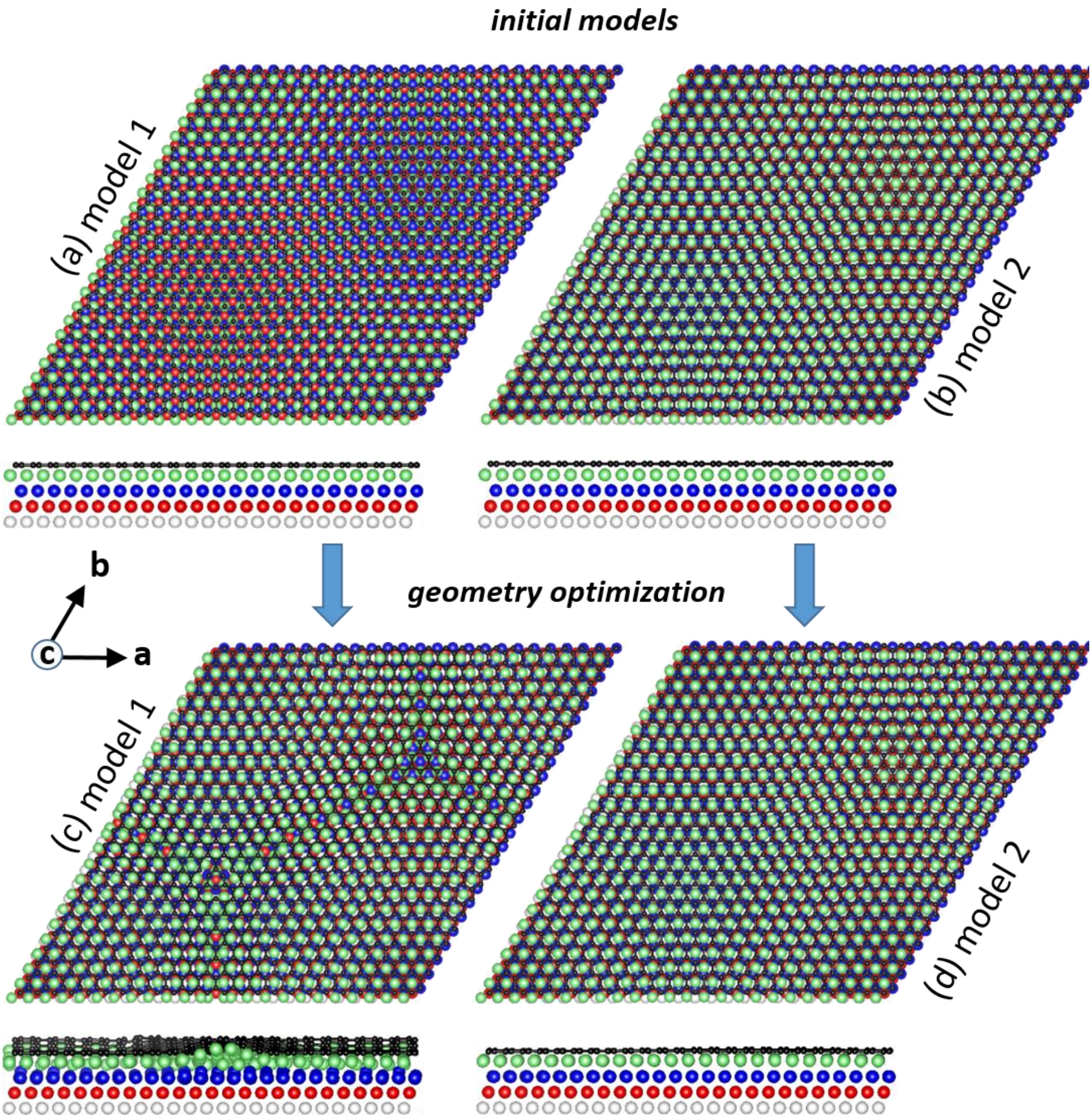


***Figure S2***: *Two possible realizations of the 24:25 supercell at the interface between honeycomb borophene and Al(111). For each model two projections are shown: along vector* ***c*** *(top) and along vector* ***a*** *(bottom). B atoms are shown in black, Al atoms in the top, second, third and fourth layers are shown in green, blue, red and white, respectively. In Model 1 (a, c) the top Al layer has 25x25 atoms in the supercell; it is lattice-matched to other Al layers but mismatched relative to the HB lattice. In Model 2 (b, d) the top Al layer has 24x24 atoms in the supercell; it is lattice-mismatched to other Al layers but matched relative to the HB lattice. (a) and (b) are initial geometries, (c) and (d) are geometries optimized by DFT calculations.*

In the case of model 1 the disturbances introduced by the lattice mismatch were so considerable that we have performed an additional calculation involving not 4 but 5 layers of Al atoms in the supercell, in order to investigate the convergence of the calculation. In both cases the bottom 2 layers were fixed in space, while the upper 2 (3) layers were allowed to relax. A comparison of the optimized geometries is shown in Figure S3. As can be seen, adding one more Al layer results in a somewhat different relaxation pattern, although the overall symmetry and level of corrugation remain similar.

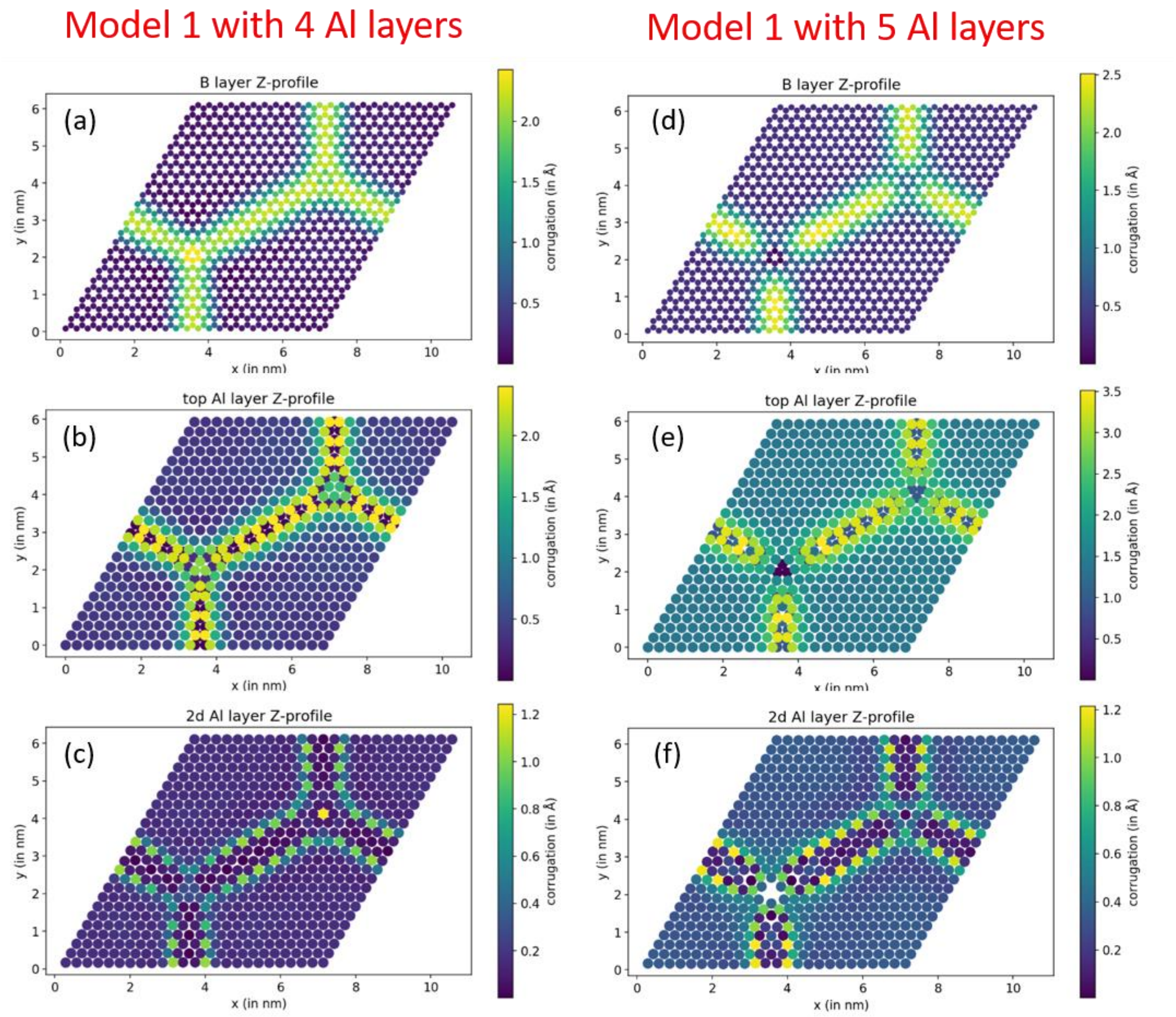


***Figure S3:*** *Layer-by-layer height profile maps for the model 1 (24x24 cells of HB on top of 25x25 slab of Al atoms) after DFT geometry optimization in the case of 4 (a-c) and 5 (d-f) layers of Al atoms included in the supercell. Profile maps are shown for the honeycomb B layer (a and d), the top Al layer (b and e) and the second Al layer (c and f).*

In analysis of the B 1s XPS spectra we performed measurements as a function of the electron emission angle in order to reveal and/or confirm the nature of individual components in the spectrum. In Figure S4 the B 1s spectrum is shown at three different detection angles. On the way to more grazing emission angles the B2 component (colored blue in Fig.S4) is gaining in relative intensity, thus providing evidence that it originates from B atoms on top of the sample.

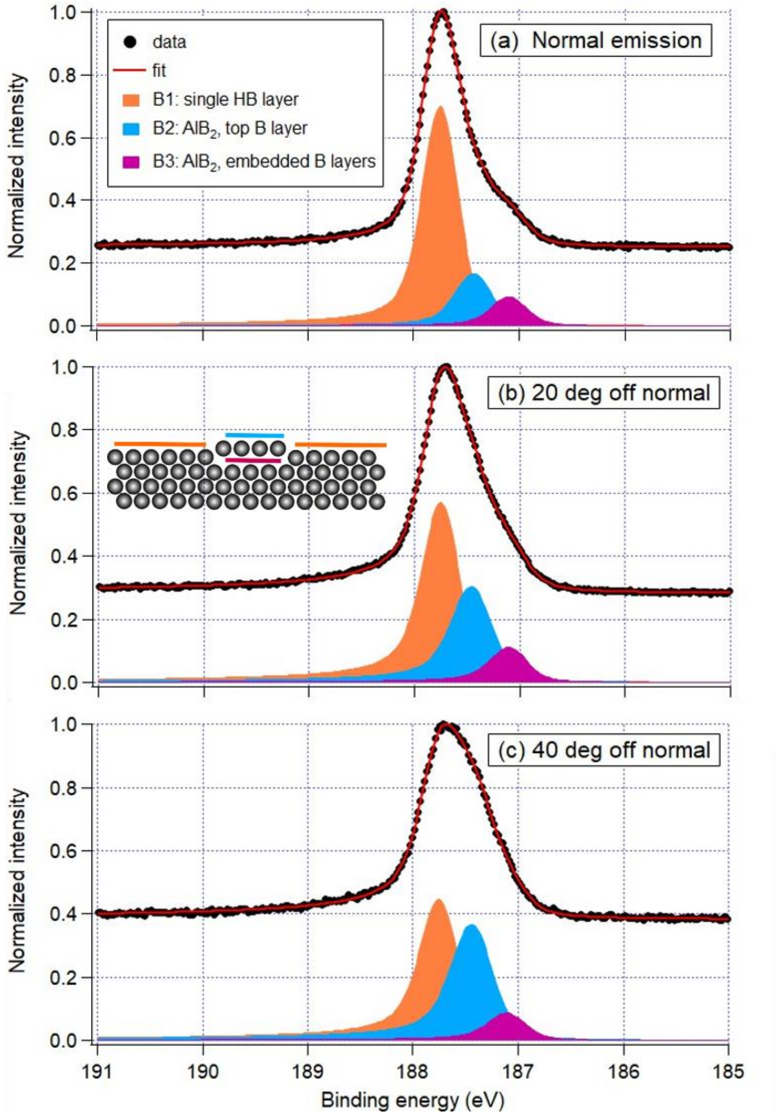


***Figure S4:*** *B 1s XPS spectra from HB on Al(111) (ca. 0.5 ML in terms of freestanding HB) taken with hν = 270 eV at different electron emission angles relative to the analyser axis: (a) normal emission (b) 20 degrees off normal and (c) 40 degrees off normal. In the schematics, grey balls represent Al atoms and coloured lines represent layers of honeycomb boron; each line colour reflects the colour of a corresponding component in the spectra.*

For analysis of PDOS in HB on Al(111) we have not only performed supercell calculations but also reproduced calculations [1, 2] with a (1 x 1) matching between B and Al lattices and B atoms located in fcc and hcp sites. As shown in the main text, the top Al layer is indeed matched 1:1 to the HB forming a stoichiometric $AlB_2$ layer, therefore this approximation is expected to be rather accurate. The result can be seen in Figure S5 (a), where PDOS curves are shown separately for B atoms in the fcc (solid lines) and the hcp (dashed lines) positions. For the sake of comparison, the outcome of the supercell calculation of boron PDOS (as in Fig.9, c of the main text) is shown in Figure S5 (b).

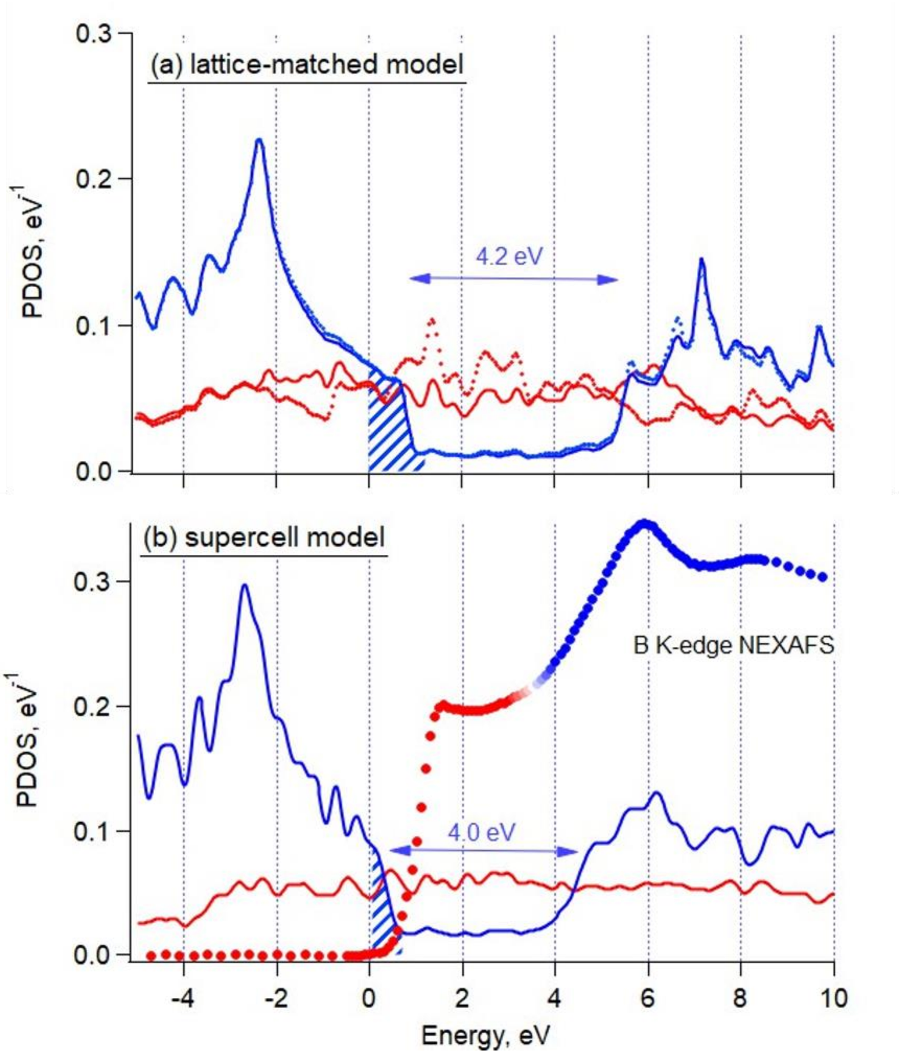


***Figure S5:*** *Calculated B 2s,p PDOS per atom for HB on Al(111). $2p_z$ (out-of-plane) PDOS curves are shown in red, $2s+2p_x+2p_y$ (in-plane) PDOS curves are blue; energy scale is relative to the Fermi level. Energy separations $\Delta E(\sigma - \sigma^*)$ are indicated by blue arrows with numbers. (a) 1:1 lattice-matched approximation (solid: fcc sites, dotted: hcp sites), (b) 24:25 supercell model. An experimental B 1s NEXAFS spectrum is shown in (b). Hatched areas indicate non-zero $\sigma$ DOS just above Fermi level.*

In the 24:25 supercell calculation there is much less σ DOS located at the Fermi level (hatched areas in Figure S4). This σ DOS has not been detected experimentally by NEXAFS.


[1] Li W, Kong L, Chen C, Gou J, Sheng S, Zhang W, Li H, Chen L, Cheng P, Wu K. Experimental Realization of Honeycomb Borophene. Sci. Bull. **2018**, 63, 282.

[2] L. Zhu, B. Zhao, T. Zhang, G. Chen, S. A. Yang. How Is Honeycomb Borophene Stabilized on Al(111)? J. Phys. Chem. C **2019**, 123, 14858.
